# Comprehensive view of macrophage autophagy and its application in cardiovascular diseases

**DOI:** 10.1111/cpr.13525

**Published:** 2023-07-11

**Authors:** Wanqian Pan, Jun Zhang, Lei Zhang, Yue Zhang, Yiyi Song, Lianhua Han, Mingyue Tan, Yunfei Yin, Tianke Yang, Tingbo Jiang, Hongxia Li

**Affiliations:** ^1^ Department of Cardiology The First Affiliated Hospital of Soochow University Suzhou China; ^2^ Suzhou Medical College of Soochow University Suzhou China; ^3^ Department of Ophthalmology, Eye Institute, Eye & ENT Hospital Fudan University Shanghai China; ^4^ Department of Ophthalmology The First Affiliated Hospital of USTC, University of Science and Technology of China Hefei China

## Abstract

Cardiovascular diseases (CVDs) are the primary drivers of the growing public health epidemic and the leading cause of premature mortality and economic burden worldwide. With decades of research, CVDs have been proven to be associated with the dysregulation of the inflammatory response, with macrophages playing imperative roles in influencing the prognosis of CVDs. Autophagy is a conserved pathway that maintains cellular functions. Emerging evidence has revealed an intrinsic connection between autophagy and macrophage functions. This review focuses on the role and underlying mechanisms of autophagy‐mediated regulation of macrophage plasticity in polarization, inflammasome activation, cytokine secretion, metabolism, phagocytosis, and the number of macrophages. In addition, autophagy has been shown to connect macrophages and heart cells. It is attributed to specific substrate degradation or signalling pathway activation by autophagy‐related proteins. Referring to the latest reports, applications targeting macrophage autophagy have been discussed in CVDs, such as atherosclerosis, myocardial infarction, heart failure, and myocarditis. This review describes a novel approach for future CVD therapies.

## Graphical abstract

This review systematically describes the role of autophagy in macrophage functions, highlighting the existing evidence in the regulation of inflammation, cell death, metabolism, phagocytosis, and so forth. Meanwhile, we summarize the latest progress of therapeutics targeting macrophage autophagy in cardiovascular diseases, hoping to provide novel approaches for future cardiac treatments.

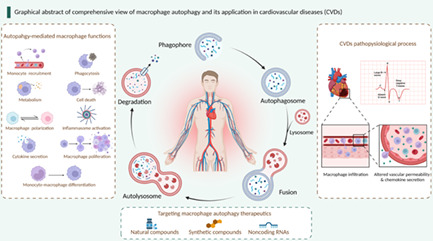

## 1 INTRODUCTION

In the 20th century, the primary causes of premature mortality and disability globally shifted from communicable, maternal, and perinatal causes to non‐communicable diseases (NCDs), with cardiovascular diseases (CVDs) serving as the dominant drivers of NCDs.^1^ Based on the American Heart Association's latest statistics, the overall CVD prevalence in the United States among adults aged ≥20 years can reach 49.2%.^2^ Senility changes the structure and function of the circulatory system. Along with related risk factors, they culminate in an enhanced burden of CVD morbidity.^3^, ^4^

Unrestrained cardiac and systemic inflammation contributes to the development of CVDs. Among the various inflammatory cells, macrophages have become a hot scientific subject with their ubiquitous presence and effects. Macrophages, which are derived from monocytes, play a wide range of roles and are highly plastic under different settings.^5^, ^6^, ^7^ In addition, macrophages play a role in the malfunctioning of endothelial cells (ECs) and cardiomyocytes, and phenotypic switching of vascular smooth muscle cells (VSMCs), all of which are strongly related to CVDs.^8^, ^9^ As a result, modulation of macrophage functions is viable targets for the CVD therapy.

Autophagy is a highly conserved biological process and a crucial nexus between physiological and pathological conditions for preserving organismal homeostasis. Perturbation of autophagy is linked to several human illnesses, particularly CVDs.^10^, ^11^ Autophagy can exert a cardioprotective effect by improving protein quality control and restricting cytotoxins. However, excessive autophagy can contribute to cardio pathology via autophagic cell death and damage.^12^, ^13^ Notably, autophagy and inflammation interact to cause CVDs, underscoring the role of autophagy in immune cells in developing heart disorders.^13^ As the primary inflammatory cells, macrophages raise extensive concern. Although macrophage autophagy has been functionally reported in atherosclerosis (AS), myocardial infarction (MI), and cardiac remodelling, no comprehensive review of macrophage autophagy in CVDs has been published. Considering the significant roles of macrophages and autophagy in CVD, we discussed their relationship and explored the potential mechanisms of autophagy‐mediated macrophage modulation to provide a novel perspective and application in CVD treatments.

## 2 THE ROLE OF MACROPHAGES IN CVDS

Chemokine receptor 2 (CCR2)‐mediated recruitment of circulatory monocytes contributes to the transendothelial accumulation of macrophages within the artery wall.^14^ Remarkably, lesional macrophage proliferation, rather than monocyte migration, is primarily responsible for replenishing macrophages in atherosclerotic plaques.^15^ Depending on the types of stimulation, macrophages will differentiate in a spectrum of polarization.^16^ Macrophages differentiate into two major subsets: classical activation type (M1‐type) macrophages stimulated by lipopolysaccharide (LPS) and interferon (IFN)‐γ, and alternatively activated type (M2‐type) macrophages stimulated by interleukin (IL)‐4 and ‐13.^16^ M1 macrophages are the leading producers of pro‐inflammatory factors, including tumour necrosis factor (TNF)‐α, IL‐1β, and C—X—C motif chemokine ligand (CXCL)‐9, with expressing surface markers, such as CD80, CD86, and inducible nitric oxide synthase (iNOS). They lead to an increase in reactive oxygen species (ROS), nitric oxide (NO), and proteolytic enzyme secretion, such as matrix metalloproteinases (MMP)‐2, ‐7, and ‐12, which break down extracellular matrix (ECM) components.^17^ Experimental evidence suggests that TNF‐α secretion from M1 may hasten aneurysm formation.^15^

In contrast, M2 macrophages release the immunosuppressive cytokines, including IL‐10, arginase 1 (Arg1), C—C motif chemokine ligand (CCL)‐18, and transforming growth factor (TGF)‐β, with expressing surface markers, such as CD206, CD209, and Fizz1. These elements promote angiogenesis, cell proliferation, and ECM component formation. However, excessive matrix proteins and collagen deposition can lead to the expansion of the cardiac infarcted size and fibrosis.^18^ Excessive TGF‐β production by M2 macrophages accelerates the synthesis of alpha‐smooth muscle actin (α‐SMA), collagen, and fibronectin in ECM, which results in non‐ischaemic‐dilated cardiomyopathy.^19^

**FIGURE 1:**
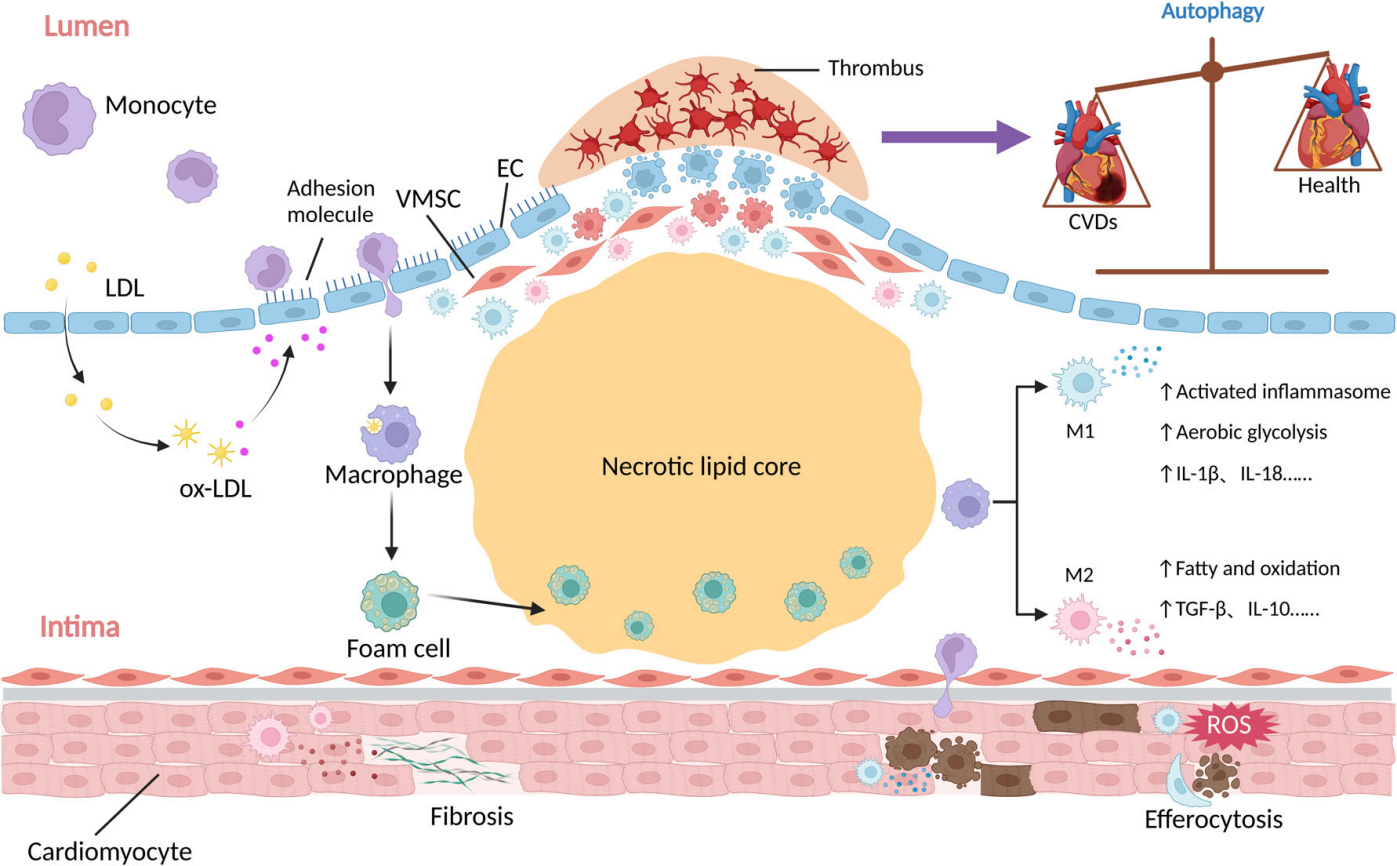
The role of macrophages in cardiovascular diseases. Low‐density lipoprotein (LDL) penetrates the endothelium and is oxidized into oxidized‐LDL (ox‐LDL), releasing pro‐inflammatory lipids. Activated endothelial cells (ECs) express adhesion molecules to govern circulating monocyte recruitment into the intima. Migrated monocytes differentiate into macrophages, which engulf ox‐LDL and shift into foam cells to enhance lipid core formation. Macrophages can exert different phenotypes and produce various cytokines, which lead to dysfunction of ECs and vascular smooth muscle cells (VSMCs) as well as dysregulation of inflammation, contributing to the development of cardiovascular diseases (CVDs). In the overall process, autophagy plays a great importance role in CVDs via regulating macrophage functions. IL, interleukin; TGF‐β, transforming growth factor‐β.

Macrophages recognize and phagocytose oxidized low‐density lipoprotein (ox‐LDL) and hydrolyse it into free cholesterol (FC) using neutral lipase. They then deliver FC to the plasma membrane via adenosine triphosphate (ATP) binding cassette subfamily A/G member 1 (ABCA1/ABCG1) and apolipoprotein (apo)‐mediated reverse cholesterol transport pathways.^20^ However, substantial ox‐LDL phagocytosis by macrophages contributes to cholesterol ester deposition, turning macrophages into foam cells and resulting in the formation the atherosclerotic plaques (Figure 1).^21^

## 3 THE ROLE OF AUTOPHAGY IN CVDS

Autophagy is a ubiquitous process by which cytoplasmic materials are degraded in the lysosome.^22^ On one hand, autophagy is induced to generate energy and building blocks by degrading the pre‐existing intracellular components and obsolete materials under physiological conditions.^23^, ^24^ On the other hand, when activated under stress conditions, such as nutrient deprivation, endoplasmic reticulum stress (ERS), and oxidative stress, autophagy eliminates abnormal protein aggregates and damaged organelles.^25^ Therefore, autophagy is crucial for maintaining renovation and homeostasis.^23^, ^24^, ^25^ Based on different mechanisms and functions, there are three forms of autophagy.^22^ Microautophagy engulfs small cytoplasmic portions via lysosomal membrane protrusions or inward invaginations. Chaperone‐mediated autophagy translocates individual substrate proteins and nucleic acids directly across the lysosomal membrane through chaperones to identify specific pentapeptide motifs. Macroautophagy (hereafter referred to as autophagy) is considered the critical method of autophagic degradation distinct from other methods because of its ability to digest macromolecules, large targets, and entire organelles through the formation of a double‐membrane compartment called the autophagosome.^10^, ^26^, ^27^

**FIGURE 2:**
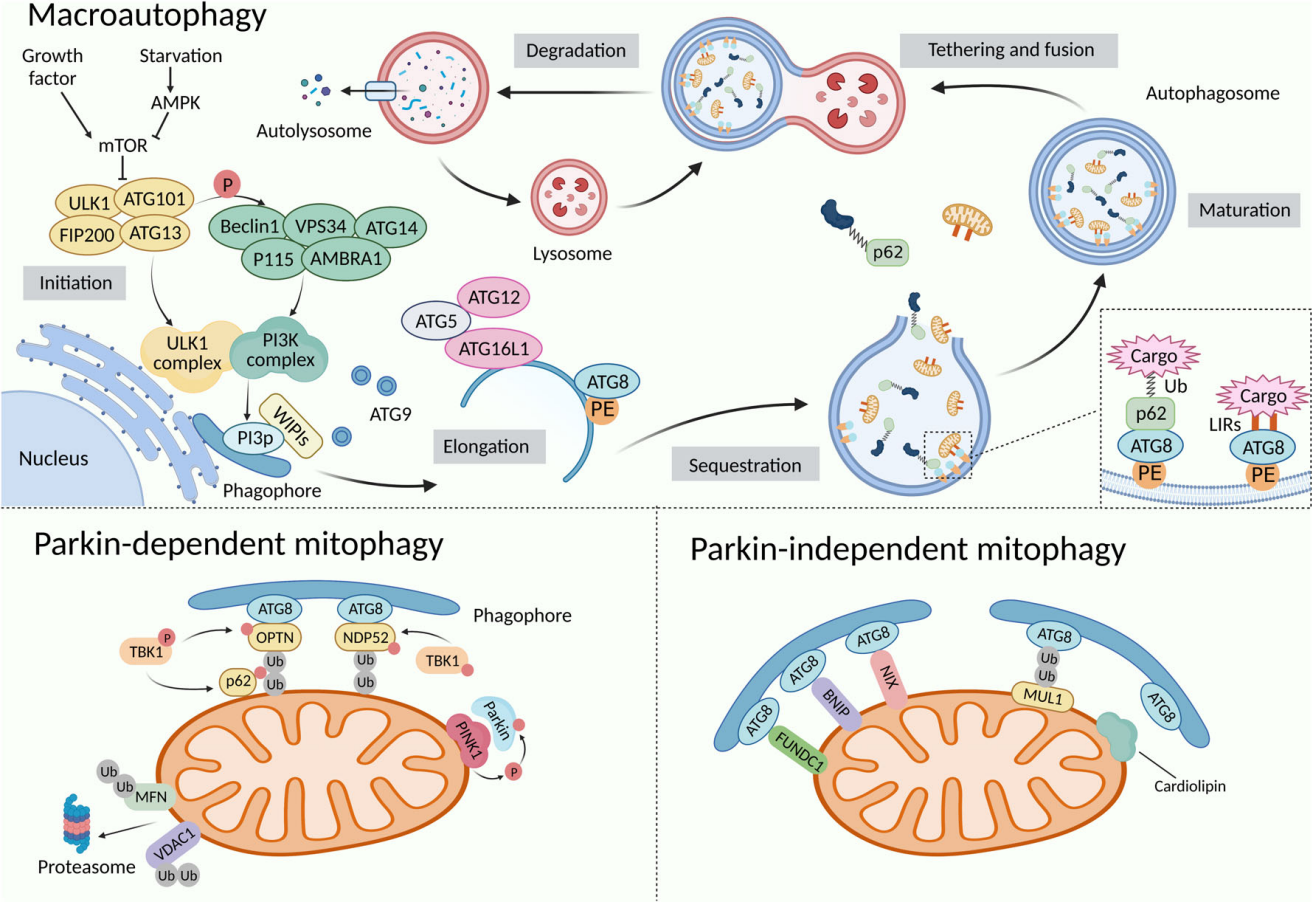
The progress of macroautophagy and mitophagy. Macroautophagy is mediated by autophagosome, the formation of which is initiated by the ULK1 complex, including ULK1, ATG101, FIP200, and ATG13. Following stimulation by amino acids and growth factors, mechanistic target of rapamycin (mTOR) is activated to hinder protein kinase complex by the phosphorylation of ATG13. But, this pathway is inhibited in a state of stress condition and low energy by activation of AMPK. The initiation complex is translocated to a particular domain of the endoplasmic reticulum. To nucleate the autophagosome membrane, ULK1 phosphorylates components of the PI3K complex, composed of Beclin1, ATG14, VPS34, P115, and AMBRA1, and generates phosphatidylinositol 3‐phosphate (PI3P) on autophagosomal precursor membrane. PI3P‐interacting WIPI family proteins (WIPIs) and ATG9‐containing vesicles are also involved. WIPIs recruit the ATG12–ATG5‐ATG16L complex and ATG8s‐PE conjugate into phagophores, essential for membrane elongation and autophagosome closure. ATG8 family proteins on the inner autophagosomal membrane are considered significant for cargo recognition directly by light chain 3 (LC3)‐interacting regions or indirectly by adaptor proteins such as p62/SQSTM1, NBR1, and TAX1BP1. After lysosome fusion, substrate proteins and the inner autophagosomal membrane are degraded to maintain intercellular homeostasis and renovation. PINK1 cannot be cleaved and forms dimers on the outer mitochondrial membrane due to the damaged mitochondrial membrane potential. This leads to Parkin recruitment and conformational changes by phosphorylating the ubiquitin‐like domain at S65. Parkin polyubiquitinates substrates as E3 ubiquitin ligases, which are recognized by autophagy adaptors to activate mitophagy, while TBK1 promotes their interaction with ubiquitin chains. In addition to cardiolipin and autophagy receptors, mitochondrial E3 ubiquitin‐protein ligase 1 (MUL1) not only plays an ubiquitination role, which has multiple common mitochondrial substrates with Parkin, but also directly participates in Parkin‐independent mitophagy as a mitochondrial receptor.

Autophagosome formation requires an orchestrated set of multiple protein complexes, especially encoded by evolutionarily conserved autophagy‐related (ATG) genes.^26^, ^28^ The Unc‐51‐like autophagy‐activating kinase (ULK)‐1 complex is critical for the induction of autophagy. It cooperates with the Class III phosphatidylinositol 3‐kinase (PI3K) complex, WD‐repeat protein interacting with phosphoinositides (WIPI), and ATG9 vesicles, and participates in the nucleation of the crescent isolation membrane, termed the phagophore, which is the initial sequestering compartment. After the elongation and enclosure of phagophore to form a spherical structure termed autophagosome, autophagosome and lysosome fuse into autolysosome to initiate autophagy activity (Figure 2).^29^, ^30^ Initially believed to be a non‐selective process that randomly degrades parts, autophagy is now known to be capable of removing specific cellular targets with precision. Depending on the type of cargo, selective autophagy can be classified into mitophagy (mitochondria), lipophagy (lipid droplets), ferritinophagy (ferritin), and so forth.^31^

Because the heart has the highest energy consumption in the body, mitochondrial activity, and mitophagy significantly affect CVDs. The autophagosomes recognize mitochondria via Parkin‐dependent and Parkin‐independent mitophagy (Figure 2).^32^ Relying on five light chain 3 (LC3) adaptors: p62, neighbour of BRCA1 gene 1 (NBR1), nuclear dot 52 kDa protein (NDP52), TAX1 binding protein 1 (TAX1BP1), and optineurin (OPTN), as the bridge connecting autophagosomes and ubiquitin‐proteasome system in the mitochondria, PTEN‐induced putative kinase 1 (PINK1) translocates Parkin from the cytosol into depolarized mitochondria and then phosphorylates and activates Parkin, which polyubiquitinates substrates and induces PINK‐Parkin‐induced mitophagy.^33^, ^34^ In Parkin‐independent mitophagy, cardiolipin and LC3 receptors, including Nip3‐like protein X (NIX), Bcl‐2/Adenovirus E1B 19 kDa interacting protein 3 (BNIP3), and FUN14 domain containing 1 (FUNDC1), are located on the outer mitochondrial membrane. They directly bind to LC3 and attract damaged mitochondria for degradation through their LC3‐interacting region (LIR) domains.^35^, ^36^, ^37^

## 4 AUTOPHAGY‐MEDIATED REGULATION IN MACROPHAGE FUNCTIONS

**FIGURE 3:**
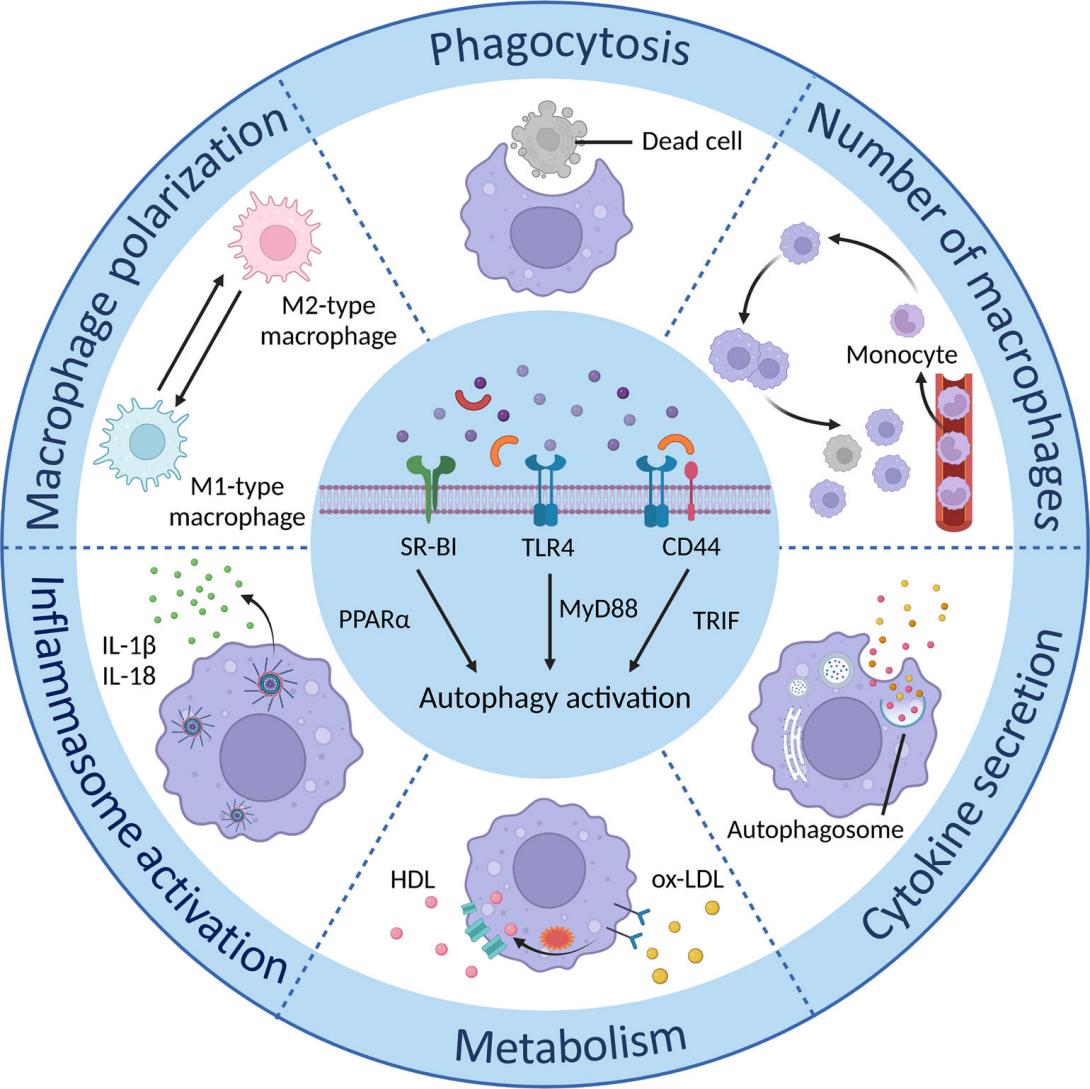
Autophagy‐mediated regulation in macrophage functions. Stimuli, such as lipopolysaccharide (LPS) and other stress factors, induce the activation of macrophage autophagy, which influences macrophage functions through various pathways, including macrophage polarization, inflammasome activation, cytokine secretion, metabolism, and phagocytosis. Changed macrophage functions subsequently affect the process and prognosis of cardiovascular diseases. HDL, high‐density lipoprotein; IL, interleukin; ox‐LDL, oxidized low‐density lipoprotein; PPAR, peroxisome proliferator‐activated receptor; SR‐BI, scavenger receptor Class B type I; TLR4, toll‐like receptor‐4.

Stimulation of toll‐like receptor (TLR)‐4 by LPS or a specific interaction with co‐receptor CD44 is reported to potentiate autophagosome formation via myeloid differentiation factor 88 (MyD88) and toll/IL‐1R domain‐containing adaptor‐inducing IFN‐beta (TRIF) signalling pathways.^38^, ^39^ Scavenger receptor Class B type I (SR‐BI), a high‐density lipoprotein receptor localized in the plasma membrane, can activate peroxisome proliferator‐activated receptor‐α to accelerate macrophage autophagy (Figure 3).^40^ The induction of autophagy can regulate macrophage activity in turn. Numerous studies have shown that autophagy can modulate macrophage functions to affect CVDs, highlighting the importance of discovering the correlation between macrophages and autophagy in cardiac diseases.^41^, ^42^, ^43^

### 4.1 Autophagy in number of macrophages

The disturbed count of macrophages is one of the characteristics of CVDs.^15^ Although there are two sources of macrophages, monocyte‐derived and tissue‐resident, the former is the primary source and is mainly responsible for uncontrolled inflammation in CVDs. In addition to monocyte recruitment, macrophages proliferation is required to maintain continuous replenishment of macrophages in lesions. Hyperlipidemia enhances macrophage proliferation, which aids in the formation of atherosclerotic plaques. Cell death also influences the number of macrophages, which triggers an immunological cascade via the release of inflammatory factors, exacerbating pathogenic alterations in CVDs. In this section, we explore the role of autophagy in regulating macrophage numbers through various steps and the underlying mechanisms based on published research (Table 1).

**TABLE 1:** Autophagy in the number of macrophages.

Category	Object	Mechanism	Reference
Monocyte recruitment	CXCL16/CXCR16 axis	Potentiate IFN‐γ secretion, autophagy activity and monocyte infiltration in ischaemic myocardium to aggravate cardiac function	47
	PCSK9	Affect autophagy and LDL release by mTOR to upregulate the level of CCR2 on monocytes	49
Monocyte–macrophage differentiation	M‐CSF	Activate CAMKK2‐AMPK‐ULK1 pathway through the accumulation of P2RY6, involving in the macrophage differentiation	60
	AMPKα1	Promote atherogenesis and lesion formation, which is associated with FOXO3‐mediated autophagy and monocyte–macrophage differentiation	61
	IL‐33	Upregulate ROS production and mitophagy via the AMPK pathway to alter the M2 polarization shift of monocytes	62
Macrophage proliferation	XBP1	Trigger macrophage proliferation via enhancing transcriptional regulation of Beclin‐1 and LC3‐II when transient activation at 24 or 48 h	67
	AMPK	Subside the expression of GM‐CSF and cell cycle arrest to impede proliferation via autophagy activation	68
Macrophage death	Stent‐based delivery of everolimus	Stabilize atherosclerotic plaque accompanied by mTOR‐dependent autophagy and autophagic cellular degradation of macrophages	71
	Berberine	Provoke TFEB deacetylation by SIRT1 to increase macrophage autophagy and diminish apoptosis	74
	Protein‐rich diets	Evoke mitophagy suppression by the phosphorylation of mTOR and mitochondrial‐dependent apoptosis to drive atherosclerotic progression	76
	Chloroquine	Elevate the p62‐Nrf2‐ARE axis to promote the pyroptosis of ox‐LDL‐treated macrophages by autophagy blockage	77
	SIRT1	Perturb excess iron‐induced ferroptosis of foam cells and pro‐inflammatory cytokine secretion by SIRT1‐autophagy axis	80

Abbreviations: AMPKα1, AMP‐activated protein kinase α1; CCR2, chemokine receptor 2; FOXO, forkhead box O; GM‐CSF, granulocyte/macrophage colony‐stimulating factor; IFN‐γ, interferon‐γ; IL, interleukin; LC3, light chain 3; M‐CSF, macrophage colony‐stimulating factor; mTOR, mechanistic target of rapamycin; Nrf2, nuclear factor E2‐related factor 2; ox‐LDL, oxidized low‐density lipoprotein; PCSK9, proprotein convertase subtilisin/kexin type 9; ROS, reactive oxygen species; SIRT1, silent information regulator 1; TFEB, transcription factor EB; XBP1, X‐box binding protein 1.

#### 4.1.1 Monocyte recruitment

Monocytes from the circulation migrate to their destinations upon stimulation by chemokines and cytokines.^15^ Among these chemoattractants, CCL‐2 is the most effective in drawing monocytes. It can extend cell longevity by suppressing caspase‐8 activation and inducing autophagy, suggesting the role of autophagy in monocyte recruitment.^44^ Consist with the discovery by Lodder et al.,^45^ autophagy inhibition in ATG5‐ablated mice elicits a higher number of recruited monocytes and exacerbates liver fibrosis.

CXCL16, a pro‐inflammatory chemokine of the C—X—C family, is positively associated with the pathogenesis of acute coronary syndrome (ACS).^46^ C—X—C motif chemokine receptor (CXCR)‐16 is a specific receptor of CXCL16. Its deficiency improves cardiac function and attenuates autophagic activity after cardiac ischemia/reperfusion (I/R) injury. Mechanical research showed that infiltration of CD11b^+^ monocytes is abated in ischaemic myocardium and concurrent IFN‐γ secretion is blocked due to the scission of the CXCL16/CXCR16 biological axis.^47^ In addition, proprotein convertase subtilisin/kexin type 9 (PCSK9) enhances the migratory capacity of monocytes, which is associated with the autophagic pathway.^48^ According to the findings, PCSK9 negatively affects autophagy by inducing the mechanistic target of rapamycin (mTOR) to inhibit the degradation of apo B, thus releasing apo B as LDLs. The subsequent accumulation of LDL upregulates the expression of CCR2 in monocytes.^49^, ^50^ In addition, many studies show that mTOR has a core relationship with monocyte migration. It not only potentiates CCL‐2 and hyaluronan deposition, a product from VSMCs that enhances monocyte adherence to the ECM,^51^, ^52^ but also provokes monocyte chemotaxis in response to diverse chemotactic stimuli.^53^ Correspondingly, atherosclerotic lesions with mTOR suppression tend to accumulate fewer macrophages.^54^, ^55^ In conclusion, autophagy plays a vital role in mediating monocyte recruitment. However, further studies are required to elucidate how autophagy modulates monocyte recruitment and to uncover the mechanisms underlying CVDs.

#### 4.1.2 Monocyte–macrophage differentiation

The half‐lives of classical and non‐classical monocytes are 1 and 7.4 days, respectively. Culturing without stimulation, they will default to a programmed apoptosis process.^56^ Once espoused to stimuli, such as ox‐LDL, 7‐ketocholesterol, and macrophage colony‐stimulating factor (M‐CSF), monocytes can initiate pro‐survival signals, migrate to distinct tissue sites, and differentiate into macrophages. Autophagy is also involved in it by specific signalling pathways.^57^, ^58^

In ATG7‐knockout mice, M‐CSF stimulation fails to differentiate monocytes into macrophages.^59^ According to mechanical research, M‐CSF accumulates P2RY6 and activates the CAMKK2‐AMPK‐ULK1 pathway, which is required for the induction of autophagy during macrophage differentiation.^60^ Furthermore, the study shows that AMP‐activated protein kinase α1 (AMPKα1) potentiates autophagic flux by activating the forkhead box O (FOXO)‐3 transcription factor. It can enhance monocyte–macrophage differentiation and survival, promoting atherosclerotic and aortic lesion formation.^61^ Treated with IL‐33, human monocyte cell line THP‐1 upregulates ROS generation with the activation of the mitochondrial respiratory chain complex, and subsequently, increases mitophagy performance by the AMPK‐dependent pathway. IL‐33 favours M2 polarization shift in monocytes; however, mitophagy scavengers mitochondrial division inhibitor‐1 (Mdivi‐1) and PINK1 gene deletion mitigate these effects.^62^ How mitophagy induces monocyte differentiation into M2‐like macrophages by IL‐33 remains to be elucidated. Further in vivo experiments are warranted.

#### 4.1.3 Macrophage proliferation

In the course of atherosclerotic lesions, intraplaque macrophage turnover is determined principally by local macrophage proliferation, not circulatory monocyte recruitment.^63^ Macrophage proliferation inhibitors can dramatically promote plaque stability at different lesion periods.^64^ Hence, reducing macrophage proliferation is an advantageous and prospective therapy for AS.

Autophagy activation may contribute to macrophage proliferation, which is related to the provision of raw materials for biosynthesis. Erwinia asparaginase can disturb macrophage autophagy, phagocytosis, and proliferation by activating Akt–mTOR and antagonizing extracellular signal‐regulated kinase 1/2 (Erk1/2) signalling pathways.^65^ Zhu et al.^66^ demonstrated that the proliferation of macrophages is attenuated dramatically when autophagy is inhibited by 3‐Methyladenine (3‐MA). Additionally, spliced X‐box binding protein 1 (XBP1) is detected at high levels in apo E^−/−^ mice, which are closely related to AS development. Transient activation of XBP1 mRNA splicing triggers autophagy via the transcriptional regulation of Beclin1 and LC3‐II, which induces macrophage proliferation.^67^ However, augmenting AMPK and autophagic flux has been demonstrated to decrease the expression of granulocyte/macrophage CSF (GM‐CSF) and cell cycle arrest to alleviate ox‐LDL‐induced primary macrophage proliferation.^68^ The network and mechanisms between autophagy and macrophage proliferation remain to be elucidated.

#### 4.1.4 Macrophage death

Macrophage death releases intricate cytokines and hydrolases that contribute to exaggerated inflammation and form a positive feedback cycle during disease development. It is widely recognized that apoptosis in macrophages, along with their defective efferocytotic function, leads to atherosclerotic plaque instability and rupture.^69^

Autophagic cell death is a form of caspase‐independent necrosis‐like cell death that is accompanied by the accumulation of autophagosomes in cells. Generally, it is considered as the decompensation and crosstalk between excessive stress and signalling pathways that regulate distinct forms of cell death via autophagy‐related particles.^70^ Stent‐based delivery of everolimus, an mTOR scavenger, stabilizes atherosclerotic plaque by selectively eliminating macrophages. It is characterized by bulk degradation of long‐lived proteins and autophagic cell death with cell shrinkage and the presence of an intact nucleus.^71^ This cell type‐specific initiation of cell death, rather than apoptosis, is the preferred type of cell death to form a stable plaque phenotype.

In the last decades, autophagy was found to participate in apoptosis. Autophagy inhibition is considered the primary cause of macrophage apoptosis. microRNA (miR)‐384‐5p‐mediated blunt macrophage autophagy induces apoptotic cell death and occurs concomitantly with aortic atherosclerotic lesions.^72^ Autophagic flux can suppress ox‐LDL‐induced apoptosis with decreased expression of caspase‐3, caspase‐9, and Bcl‐2 associated X (Bax) in atherosclerotic plaques, which influences the vulnerability of plaques and progression of atherogenesis.^73^ Research has found that Berberine treatment can enhance silent information regulator (SIRT)1‐transcription factor EB (TFEB)‐mediated autophagy via NAD^+^ synthesis pathway to mitigate macrophage apoptosis.^74^

In combination with caspase activation, the impaired mitochondrial ultrastructure, mitochondrial fragmentation, and excessive ROS generation synergistically contribute to apoptosis. Autophagy antagonizes oxidative stress damage by diminishing mitochondrial dysfunction and non‐canonical nuclear factor‐kappaB (NF‐κB).^75^ Zhang et al.^76^ innovatively discovered that protein‐rich diets elevate the level of leucine and deteriorate macrophage apoptosis. Leucine synergizes with atherogenic lipids to induce the phosphorylation of mTOR and subsequent mitophagy inhibition, contributing to the accumulation of dysfunctional mitochondria and mitochondria‐dependent apoptosis in intraplaque macrophages.

Furthermore, autophagy can alter other cell death pathways, such as pyroptosis and ferroptosis. Synergizing with ox‐LDL, chloroquine (CQ) blocks autophagic flux to activate the nuclear factor E2‐related factor 2 (Nrf2)‐ARE signalling pathway by the accumulated p62. Then enhanced Nrf2 aggravates the excessiveness of p62, exacerbating autophagy damage and pyroptosis with upregulated levels of gasdermin D (GSDMD) and lactate dehydrogenase (LDH).^77^ Electrical stimulation (ES), a non‐invasive and secure approach, has been found to heal and regenerate tissues to promote symptoms in health conditions. Macrophages subjected to ES evoke autophagosome maturation and autophagy activity by potentiating SIRT‐3. Subsequently, it leads to the deacetylation of ATG5, which nullifies ROS generation and ameliorates pyroptosis.^78^ Considering mitochondria are the primary site of ROS production and SIRT3, the co‐localization of mitochondria and autophagy markers helps verify whether mitophagy is involved.

Iron deposition and ferroptosis play pivotal roles in plaque formation and dysfunction. Impaired autophagy triggers ferroptosis with a decline in glutathione peroxidase 4 levels in THP‐1 macrophage‐derived foam cells, which is attributed to the padlock of Nrf2‐manipulated antioxidant defence and initiation of intracellular ROS generation.^79^ Su et al.^80^ discovered that excess iron induces foam cell ferroptosis, lipid ROS level, and consequent IL‐1β and IL‐18 production, which is blocked by the SIRT1‐dependent autophagy axis. Similarly, autophagy activator rapamycin (RAP) restores the detrimental effects of high uric acid levels on foam cell ferroptosis.^81^ One presumption is that a limited amount of autophagic flux will alleviate the level of ROS in cells and prevent ferroptosis. In contrast, the excessive activation of autophagy may digest ferritin and elevate iron levels to encourage ferroptosis. Although autophagy is an essential regulator of cell death, its two‐way function should be noted.

### 4.2 Autophagy in macrophage polarization

Differently polarized types of macrophages play a role in CVDs by influencing cardiac inflammation. M1 macrophages aggravate injury by releasing pro‐inflammatory factors, whereas M2 macrophages are involved in wound repair and ECM reconstruction. Wang et al.^82^ demonstrated that the autophagy‐related gene, *HSPB8*, is involved in macrophage polarization in AS. M2 macrophages are the predominant subtype in the low HSPB8 group. Furthermore, Tanshinone IIA has a propensity to M2 polarization by reestablishing the interaction between KLF4 and MCPIP, which facilitates the ERS‐dependent autophagy and activation of signal transducer and activator of transcription (STAT)‐6.^83^ Altogether, these findings indicate a potential correlation between autophagy and macrophage polarization.

SIRT1 and mTOR may be crucial factors in autophagy and macrophage polarization. Gypenoside (GP) and *Laminaria japonica* polysaccharide (LJP61A) restore ox‐LDL‐induced autophagy impairment to attenuate M1 polarization and AS by the SIRT1‐FOXO1‐mediated pathway.^84^, ^85^ Araloside C, a natural saponin, exerts an anti‐inflammatory effect in AS with tendentious M2 polarization by boosting autophagic flux in a SIRT1‐dependent pathway.^86^ In addition, 5‐aminolaevulinic acid‐mediated non‐lethal sonodynamic therapy provokes a shift in M1/M2 equilibrium toward M2 via prompting the ROS‐AMPK‐mTOR‐autophagy signalling pathway.^87^ Atheroma lesions treated with rosuvastatin (RVS) have a lower lipid profile and necrotic core. Due to the suppression of PI3K‐Akt‐mTOR, RVS can improve autophagy initiation, enhancing M2 polarization conversion, and cholesterol efflux.^88^

**FIGURE 4:**
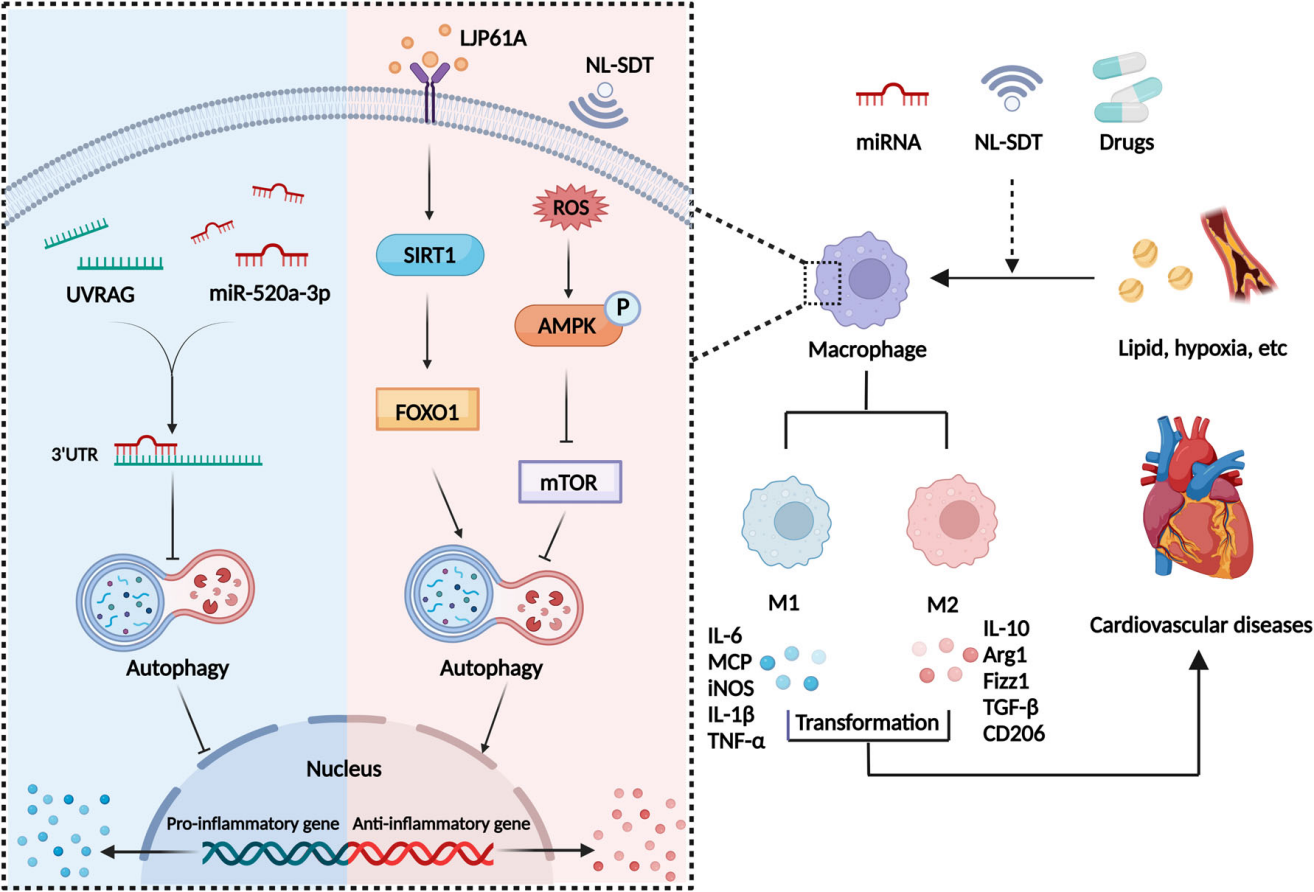
Autophagy in macrophage polarization. miRNA‐mediated epigenetic regulation controls autophagic activity through RNA‐targeted binding to molecules upstream of autophagy, leading to the alternation of macrophage polarization. Furthermore, the activation of the SIRT1‐FOXO1 signalling pathway and application of non‐lethal sonodynamic therapy (NL‐SDT) improve autophagy defect and subsequently favour a shift in M1/M2 equilibrium toward M2. Modification in macrophage polarization can impact the local inflammatory microenvironment, thereby regulating the pathophysiology and development of cardiovascular diseases. Arg1, arginase 1; IL, interleukin; iNOS, inducible nitric oxide synthase; MCP, monocyte chemoattractant protein; mTOR, mechanistic target of rapamycin; TGF‐β, transforming growth factor‐β; TNF‐α, tumour necrosis factor‐α.

miRNAs also participate in macrophage polarization by altering autophagy. miR‐29a can increase autophagic activity and promote M2 differentiation to ameliorate AS.^89^ Forced expression of miR‐520a‐3p dampens the expression of LC3 and degradation of p62 to retard IL‐4‐triggered M2 differentiation through the interaction with 3′ untranslated region of UVRAG to accelerate the progression of AS (Figure 4).^90^

Notably, the cell composition of the plaques is intricate at different stages. Macrophages predominate during early atherosclerotic plaques; however, foam cells take over in the late stages and transition from macrophages by phagocytizing lipids. Sun et al.^91^ found that RAP, an mTOR inhibitor, induces autophagic flux with upregulated LC3 in both peritoneal macrophages and foam cells. However, it augments the expression of IL‐6 and COX2 (M1 markers) and reduces lipid droplet accumulation in peritoneal macrophages only. Conversely, fingolimod permits foam cells to undergo autophagy‐mediated M2 polarization to ameliorate advanced AS. As a result, the atheroprotective benefits vary depending on the autophagy modulators used for each cell type.

### 4.3 Autophagy in inflammasome activation

Inflammasomes comprise a cytosolic sensor protein (either a nucleotide‐binding domain and leucine‐rich‐repeat‐containing [NLR] protein or an AIM2‐like receptor [ALR] protein), an adaptor protein (apoptosis‐associated speck‐like protein containing a CARD [ACS]), and an effector pro‐caspase‐1.^92^ Assembly of inflammasome leads to autocatalytic cleavage of an inactive pro‐caspase‐1 into an active caspase‐1, which regulates the proteolytic maturation of IL‐1β and IL‐18 in turn.^93^ Activated caspase‐1 cleaves GSDMD to cause a pyrogenic, inflammatory form of programmed cell death called pyroptosis.^92^

Dysregulation of inflammasome activation accelerates the pathogenesis of CVDs, attributed to the undesirable inflammatory response.^94^ Increased autophagic flux can subside NLRP3 inflammasome activation and subsequent macrophage pyroptosis.^95^ TFEB evokes atheroprotection via autophagy‐lysosome system, suppressing the inflammasome and cytotoxic protein aggregates.^96^ Additionally, an evident reduction in IL‐1β, IL‐18, and NLRP3 inflammasome is observed in arglabin‐treated macrophages concomitantly with increasing clustering of LC3 at the autophagosomal membrane.^97^

Autophagy may hinder inflammasome activity by degrading inflammasome constituents. This depends on the increased amount of the autophagy adaptor p62 that co‐immunoprecipitates with ASC.^98^ Remarkably, chaperone‐mediated autophagy is also involved in the progression of AS. Its impairment promotes NLRP3 inflammasome activation, predominantly in macrophages, among the cell types in the plaques. The NLRP3 protein, but not ASC or pro‐caspase‐1, interacts with heat shock cognate 71 kDa protein (HSC70) and lysosome‐associated membrane protein type‐2A (LAMP‐2A) to be degraded by chaperone‐mediated autophagy.^99^ Besides, precision autophagy can selectively mar inflammasome components. Previous reports have stated that NLRP3 is explicitly degraded by the tripartite motif‐containing (TRIM)‐20 and E3 ubiquitin ligase MARCH7.^100^, ^101^

Mitochondrial dysfunction directly activates NLRP3 inflammasome by inducing K^+^ efflux and releasing damage‐associated molecular patterns (DAMPs), such as mitochondrial DNA (mtDNA) and mitochondrial ROS (mtROS).^102^ Shen et al.^103^ reported that peritoneal macrophages from LDLR^−/−^ mice decrease IL‐1β production and caspase‐1 cleavage to reduce AS when fed dietary polyunsaturated fatty acids (PUFA). The mechanistic study discovered that PUFA‐enriched diets potentiate autophagic activation and mitigate dysfunctional mitochondria. Mitophagy phagocytizes the damaged and dysfunctional mitochondria to control inflammasome activity.^104^ Melatonin‐mediated mitophagy suppresses NLRP3 inflammasome activation in atherosclerotic lesions via the SIRT3‐FOXO3a‐Parkin signalling pathway.^105^ Synergizing with DNase II, mitophagy limits mtDNA damage and degrades ROS‐producing mitochondria to reduce LPS‐induced NLRP3 inflammasome expression in THP‐1 and primary macrophages.^106^ In contrast to these findings, Liu et al.^107^ indicated that autophagy could activate NLRP3 inflammasome in MI. An autophagy specific‐inhibitor, 3‐MA attenuates IL‐1β production and NLRP3 inflammasome formation to improve cardiac function and fibrosis. However, further studies are required to elucidate the underlying mechanisms by which autophagy upregulates NLRP3 activation in MI.

**FIGURE 5:**
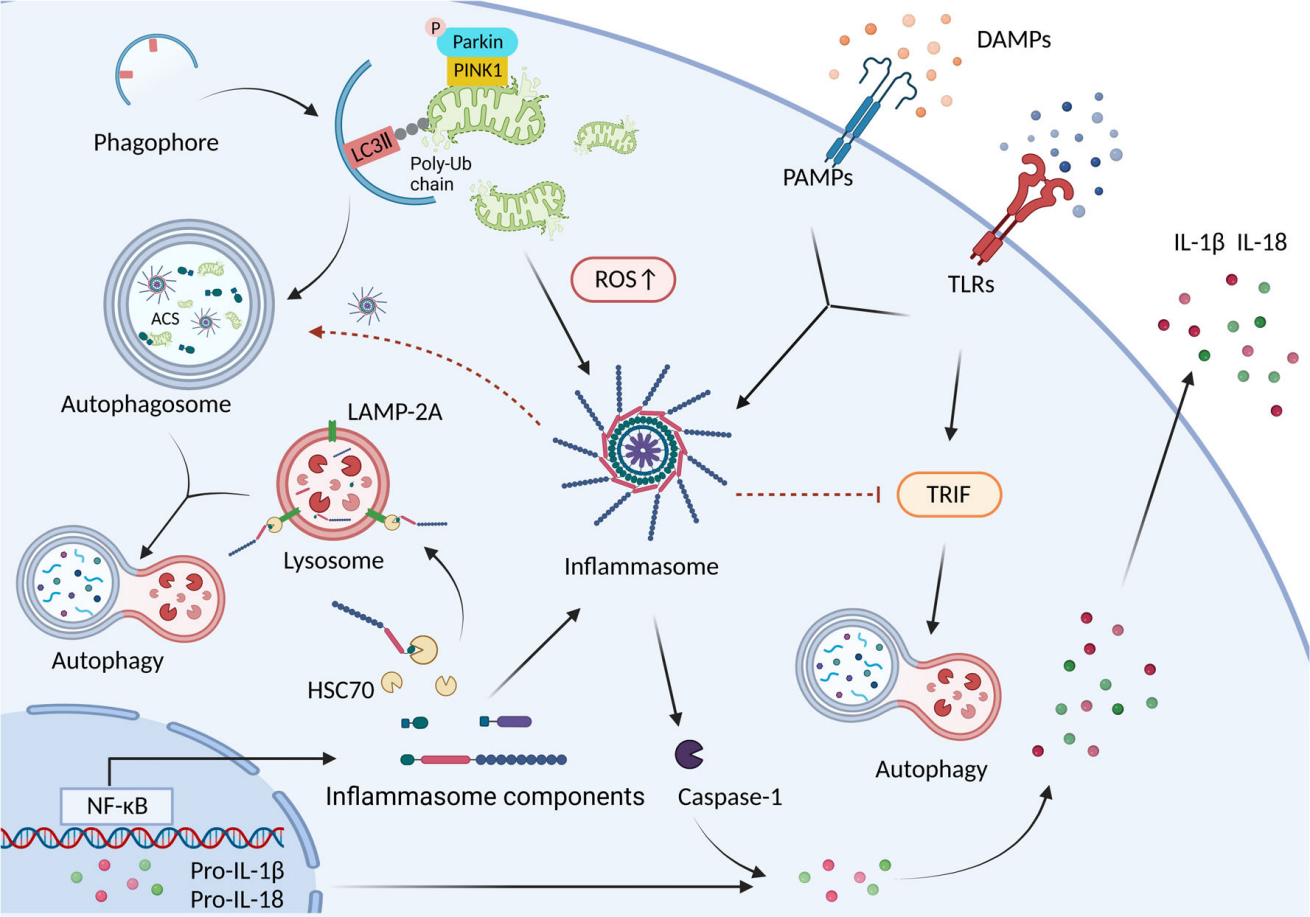
Autophagy in NLRP3 inflammasome activation. The formation of NLRP3 inflammasome requires two steps: priming and triggering. nuclear factor‐kappaB (NF‐κB) pathway is activated to increase the transcription and translation of NLRP3 inflammasome components and related cytokines as the priming signal. This process is initiated by damage‐associated molecular patterns (DAMPs), which stimulate pathogen‐associated molecular patterns (PAMPs), toll‐like receptors (TLRs), and downstream factors. The damaged mitochondria trigger NLRP3 inflammasome activation. Increased autophagic flux can abrogate the impaired depolarized mitochondrial and mitochondrial reactive oxygen species (mtROS) production through mitophagy. In addition, autophagosome directly engulfs ACS to attenuate its polymerization, thus limiting the activation of NLRP3 inflammasome and associated inflammation. Notably, chaperone‐mediated autophagy is also involved in regulating inflammasome via the degradation of NLRP3 protein. NLRP3 inflammasome can modulate autophagy in turn. The interaction between NLRP3 inflammasome and autophagy is associated with active caspase‐1, which cleaves toll/IL‐1R domain‐containing adaptor‐inducing IFN‐beta (TRIF) to inhibit TLR4‐TRIF‐mediated activation of autophagy. IL, interleukin.

Inflammasomes also participate in the regulation of autophagy function. The activation of AIM2 and NLRP3 inflammasomes can induce autophagy, which does not require ASC, caspase‐1, IL‐1β, or complete assembly of inflammasome; however, it depends on the presence of the cytosolic sensor protein.^98^ NLRP3 inflammasome antagonizes TLR4‐TRIF‐mediated autophagy in microglia. It generates active caspase‐1 to cleave the signalling protein TRIF.^108^ Notably, the TLR4‐TRIF pathway also stimulates autophagy in macrophages.^39^ As a result, the interaction between autophagy and inflammasomes is closely related but complex, requiring more studies to better understand the distinct mechanisms (Figure 5).

### 4.4 Autophagy in cytokine secretion

Cytokine production by macrophages, such as IL‐1, IL‐6, IL‐10, IL‐18, and TGF‐β, orchestrates various immune regulations in CVDs. The conventional and unconventional secretion pathways are two types of cytokine secretion pathways. In the conventional secretion pathway, cytokines with specific signal peptides facilitate endoplasmic reticulum (ER) targeting. They are then translocated into the Golgi and delivered to the cell plasma membrane for extracellular secretion through secretory vesicles.^109^ Previous studies have found that autophagy processes affect the conventional secretion of IL‐6 and IL‐8 through the TOR‐autophagy spatial coupling compartment (TASCC), a specialized compartment and lysosomal‐like organelle juxtaposed to the Golgi.^110^ Autophagosomes wrap and deliver cargo to the TASCC for degradation. It simultaneously produces amino acids and other metabolites to promote the activation of TASCC‐resident mTOR and provides essential building blocks for the extracellular translation and secretion of IL‐6 and IL‐8.^111^ HL60 human promyelocytic leukaemia cells differentiate into macrophage‐like cells after stimulation with 12‐O‐tetradecanoylphorbol‐13‐acetate, accompanied by transient production of IL‐8 and activation of autophagy. A substantial population of attached cells exerts local enrichment for mTOR, lysosomes, and autophagy vacuoles in the perinuclear regions.^112^

Cytokines deficient in leader peptides cannot reach the ER and are secreted by an unconventional pathway where autophagy takes part.^113^ Stimulation of autophagy by starvation contributes to the unconventional secretion of IL‐1β in macrophages, which depends on ATG5, Golgi reassembly stacking protein 55 (GRASP55/GORASP2), and Ras‐related protein (Rab)‐8a.^114^ According to mechanical research, specific TRIM proteins and soluble *N*‐ethylmaleimide sensitive factor attachment protein receptor (SNARE) proteins are responsible for the unconventional secretion route in bone marrow monocytes. IL‐1β is recognized by TRIM16 and translocated to the LC3‐II packing carrier when TRIM16 combines with Sec22b (a protein involved in vesicle trafficking). In conjunction with synaptosomal‐associated protein 23/29 (SNAP23/29) and syntaxin3/4 (STX3/4), Sec22b molecules in autophagosomes fuse with the plasma membrane to deliver IL‐1β for extracellular secretion.^115^ However, none of these experimental findings have been confirmed in animal studies, and it is debatable whether they are involved in regulating macrophage cytokine secretion in CVDs. Conversely, autophagy can suppress IL‐1β expression by removing inflammasomes and pro‐IL‐1β.^116^ Targeted delivery of RAP perturbs IL‐1β production to reverse plaque inflammation and burden via mTOR‐SIRT1‐mediated restriction of NF‐κB.^117^ It suggests that autophagy may act as a double‐switch mechanism that promotes IL‐1β secretion for defence during the early phase of stress conditions while inhibiting IL‐1β synthesis to avoid over‐inflammation during the late stages.

Additionally, the role of autophagy in cytokine secretion extends to the unconventional pathways of TGF‐β1, IL‐18, and the DAMP HMGB1.^114^, ^118^ TGF‐β1 interacts with GRASP55/GORASP2 in the Golgi and is secreted by Golgi‐derived microtubule‐associated protein 1 light chain 3 (MAP1LC3)‐positive autophagosomal intermediates.^118^ TGF‐β1 is the first cytokine that contains a signal peptide to enter the Golgi apparatus for maturation and proper folding, but still needs an autophagic pathway for secretion. This innovative study could be utilized for the development of anti‐myocardial fibrosis strategies.

Apart from IL‐1β and IL‐18, IL‐1α is also delivered to the cellular membrane by autophagosome‐mediated unconventional secretion when caspase‐1 is activated.^110^ A calpain‐dependent process stimulated by mitochondrial uncoupling also involves the activation and production of IL‐1α.^119^ Therefore, mitophagy can downregulate both the initiation of inflammasomes and calpain pathway, leading to the alleviative secretion of IL‐1α. Furthermore, the explicit block of PI3K‐Akt‐mTOR signalling pathway encourages macrophage autophagy. It reduces atherosclerotic plaque susceptibility and lipid load with decreased IL‐10 and increased IFN.^120^ These findings broaden the functional manifestations of autophagy beyond its autodigestive role as a more widespread process in cytosolic protein extracellular delivery.

### 4.5 Autophagy in metabolism

Emerging reports emphasize the predominant role of metabolic reprogramming in the diversity and plasticity of macrophages, also known as immunometabolism.^121^ Macrophages can alter their metabolic programmes in response to stimuli to meet the specialized needs of host defence, tissue repair, and homeostasis. Aerobic glycolysis and the pentose phosphate pathway are integral to M1 polarization and function. Upregulation of glycolysis can maintain ATP production, acidize the environment, and provide precursors for lipid biosynthesis.^122^ Whereas M2 macrophages use the fatty acid oxidation (FAO) pathway and enhance mitochondrial biogenesis to produce ATP.^123^ Fatty acid synthesis (FAS) modifies macrophage adhesion, recruitment, and inflammatory initiation by organizing plasma membranes.^122^ Aberrant accumulation of intercellular lipid causes macrophages to activate ERS and NLRP3 inflammasome activation.^124^ This reveals the close relationship between metabolic reprogramming and divergent macrophage activities.

mTOR and AMPK, two principal autophagy regulators, have been used to probe the relationship between metabolic status and the autophagy machinery.^122^, ^123^ mTOR can induce protein synthesis, cap‐dependent translation, and ribosomal biogenesis under amino acid abundance. Macrophages isolated from the patients with IL‐10 receptor mutations display a pro‐inflammatory response. Mechanistic study suggested that IL‐10 inhibits the activation of mTOR by STAT3‐DDIT4 pathway, which promotes mitophagy and oxidative phosphorylation and hinders aerobic glycolysis.^125^ mTOR signalling pathway supports the transition toward glycolysis required by M1 macrophages via elevating HIF‐1 expression. It results in the upregulation of glycolytic enzymes and pro‐inflammatory genes.^126^ mTOR catalyses fatty acids and triacylglycerol synthesis, while reducing lipolysis and FAO. It also exacerbates intracellular lipid accumulation via sterol regulatory element‐binding protein, which codes for the expression of lipogenesis‐associated enzymes.^127^, ^128^ AMPK, as a primary sensor of cellular energy status, enhances FAO and suppresses FAS, protein biogenesis, and other anabolic programmes to meet the demands of ATP.^123^ AMPK encourages FAO to mitigate lipid intermediates and inflammation via antagonizing acetyl‐coA carboxylase1, 3‐Hydroxy‐3‐methylglutaryl‐CoA reductase, and augmenting ULK1‐dependent mitophagy.^124^, ^129^

**FIGURE 6:**
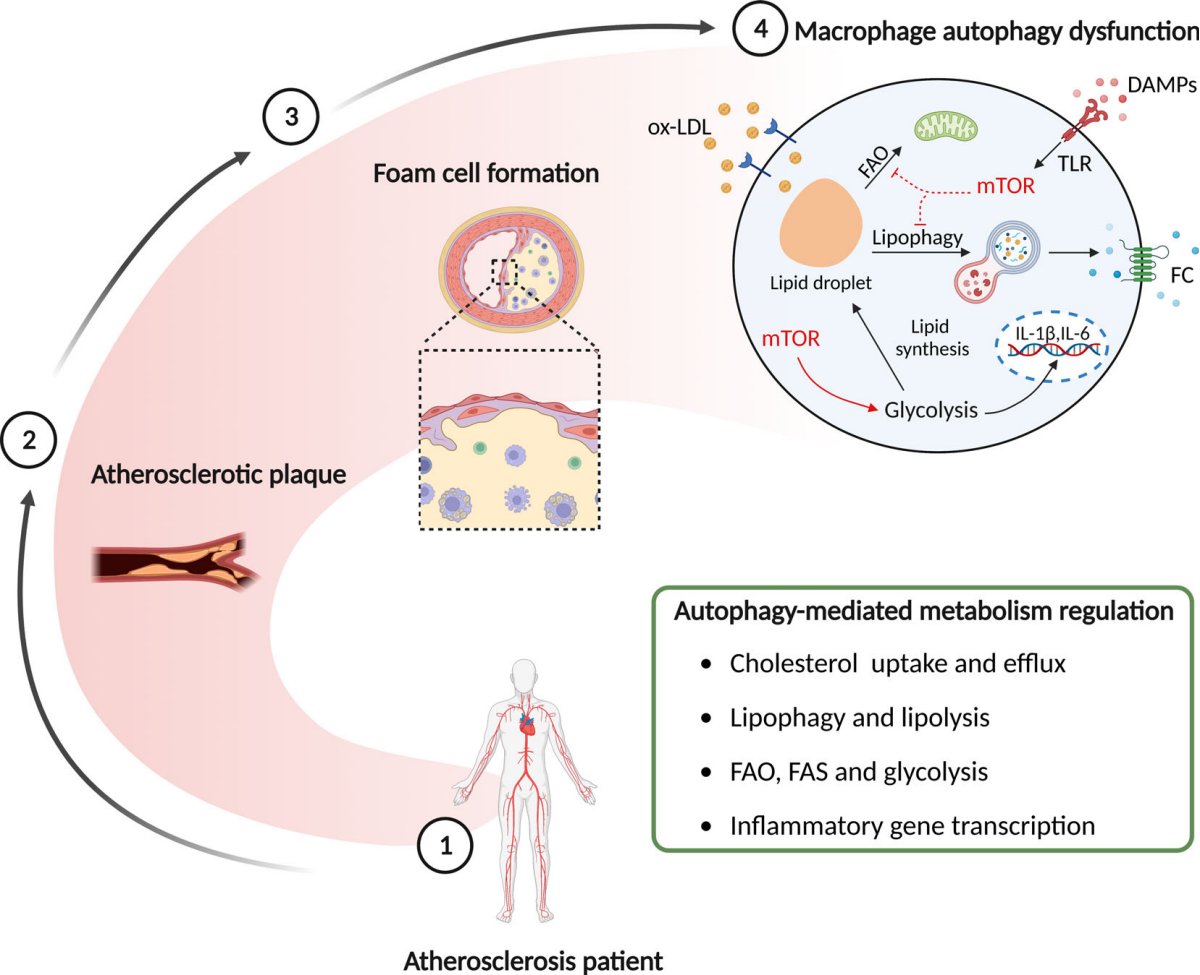
Autophagy in metabolism. In terms of lipid metabolism, mechanistic target of rapamycin (mTOR) facilitates glycolysis to upregulate lipid syntheses and pro‐inflammatory gene transcription, such as interleukin (IL)‐1β and IL‐6. And, the progression of AS is exacerbated under mTOR‐mediated suppression of lipophagy and fatty acid oxidation (FAO). While AMP‐activated protein kinase (AMPK) and other autophagy agonists can impact macrophage metabolism via disparate manners, such as cholesterol uptake, lipophagy, and fatty acid synthesis (FAS). FC, free cholesterol; ox‐LDL, oxidized low‐density lipoprotein; TLR, toll‐like receptor.

In cooperation with neutral lipase, autophagy hastens the hydrolysis of lipids to FC and move out of macrophages to ameliorate plaque stability.^130^ Ginsenoside Rb1 increases lipolysis to mitigate lipid accumulation in foam cells by inducing AMPK phosphorylation and autophagy activation.^131^ miR‐33, a post‐transcriptional regulator of genes involved in cholesterol homeostasis, targets essential autophagy effector genes (*ATG5*, *TFEB*, *ATG12*, and *ATG4b*) and AMPK‐activated transcription factors (FOXO3 and Tcfeb) to reduce lipid droplet catabolism. Additionally, miR‐33 inhibits the clearance of apoptotic cells and the digestion of their cargo by an autophagy‐dependent mechanism, which aggravates the pathological aspects of AS (Figure 6).^132^ In addition, cordycepin and ursolic acid have been demonstrated to promote macrophage autophagy and cholesterol efflux, exerting anti‐atherosclerotic effects.^133^, ^134^ A recent study found that Ca^2+^‐calpain signalling is a pivotal upstream of autophagy. LJP61A markedly reduces intracellular Ca^2+^ to provoke macrophage autophagy, resulting in the suppression of lipid accumulation and AS.^135^

Apart from increasing lipophagy, autophagy can also regulate the ABCA1/ABCG1 transport pathway to alleviate AS. Liang et al.^136^ reported that activation of autophagy enhances the cholesterol trafficking out of foam cells by promoting the expression of ABCA1, ABCG1, and their upstream transcription factor LXRα. However, the impairment of autophagy inhibits FC delivery extracellularly by p62‐mTOR‐LXRα pathway. A reduction in ox‐LDL ingestion abrogates the accumulation of cholesterol ester to relieve AS. ox‐LDL is identified and engulfed into macrophages by scavenger receptors, such as LOX‐1, class A scavenger receptor (SRA), and CD36.^130^ According to the study, enhanced autophagy significantly retards ox‐LDL uptake, foam cell formation, and migration by autolysosome‐dependent CD36 degradation at the post‐transcriptional level.^137^

### 4.6 Autophagy in phagocytosis

Phagocytosis is defined as the pathway for the recognition and internalization of particles (>0.5 μm). Through endocytosis of soluble ligands, targets are engulfed within the plasma membrane envelope of phagocytes.^138^ Engulfment of exogenous particles results in the maturation of single‐membrane phagosome vacuoles. They subsequently fuse with lysosomes and promote acidification, culminating in the formation of phagolysosomes for digestion.^138^ Apart from microbial particles, phagocytosis can remove cellular corpses and debris, predominantly by macrophages, in a process known as efferocytosis. They can recycle cellular components and mitigate inflammation in the local tissue milieu. These processes are essential for the anti‐inflammation and removal of apoptotic cells in plaques and dead cardiomyocytes during MI.^139^ Advanced atherosclerotic lesions are characterized by impaired efferocytosis.^140^

**FIGURE 7:**
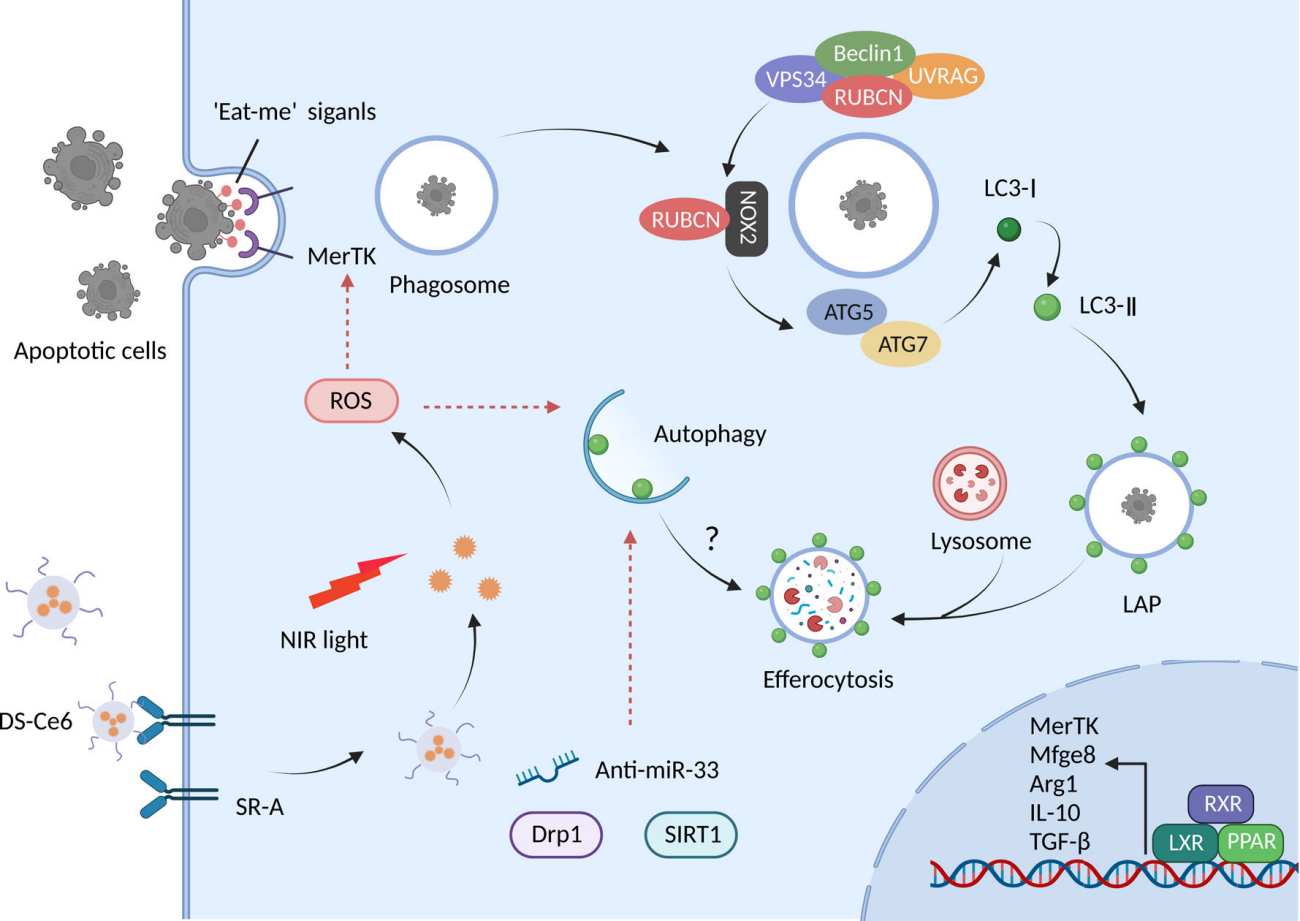
Autophagy in phagocytosis. Macrophages employ receptors that recognize ‘eat me’ signals to uptake dead cells. The engulfment of cellular corpses triggers the recruitment of the PI3K complex to form LAPosome. The interaction of Rubicon (RUBCN) and NOX2 provokes the generation of reactive oxygen species (ROS) required to recruit ATG12–ATG5‐ATG16L and ATG8s‐PE conjugation systems. To initiate light chain 3 (LC3)‐associated phagocytosis (LAP), the decoration of LAPosome is essential for the fusion of the lysosomal network. The photo‐theranostic agent DS‐Ce6 enhances autophagy and recognition receptor Mer tyrosine kinase (MerTK) expression within macrophages, improving lesion efferocytosis. Concurrently, phagocytes will activate retinoid X receptors (RXR), LXR, and peroxisome proliferator‐activated receptor (PPAR) pathways and downstream molecules to initiate the ‘tolerate‐me’ signalling and immunotolerant process. Arg1, arginase 1; IL, interleukin; MerTK, Mer tyrosine kinase; TGF‐β, transforming growth factor‐β.

Autophagy plays a favourable role in AS and abates its progression by promoting the efferocytosis of apoptotic cells. Dynamin‐related GTPase1 (Drp1)‐deficient macrophages subside mitophagy and render efferocytosis defective, contributing to atherogenesis and aortic aneurysms.^141^ Macrophage‐targeted photoactivation, a promising therapeutic strategy for CVDs, promotes macrophage autophagy activation with the specific affinity binding to the scavenger receptor. Mechanistic results show that imaging‐guided DS‐Ce6 light activation dampens plaque burden and inflammation owing to the elevation in apoptotic cell engulfment via increasing autophagy and phagocytic receptor Mer tyrosine kinase (MerTK) expression in foam cells (Figure 7).^142^ Moreover, resveratrol enhances SIRT1‐mediated autophagy and improves the defective efferocytosis of ox‐LDL‐induced apoptotic cells.^143^ PUFA‐mediated atheroprotection is associated with the arrest of caspase‐1 cleavage, apoptosis, necrosis, and amelioration of efferocytosis, whereas myeloid ATG5 deletion abolishes these protective effects.^144^ Additionally, TEFB, an essential autophagy molecule, facilitates phagocytosis‐related genes and impedes lesion complexity.^145^ These findings indicate that the activation of macrophage autophagy‐mediated efferocytosis may serve as a cutting‐edge approach for treating AS.

In 2007, Sanjuan et al.^146^ first reported that activation of the TLR pathway promotes LC3 conjugation with the phagosome membrane in macrophages requiring LC3, ATG7, and Beclin1 activity, defined as LC3‐associated phagocytosis (LAP). Distinct from canonical autophagy, LAP is independent of the ULK1 complex for phagophore formation under nutrient deprivation or stress stimulation; however, it utilizes a portion of key canonical autophagy proteins. The PI3K complex is necessary for both autophagy and LAP. The core components of the PI3K complex are Beclin1, VPS15, VPS34, UVRAG, and Rubicon during LAP without ATG14 and Ambral, which are the two indispensable subunits of autophagy. Furthermore, ubiquitin‐like systems, including ATG3, ATG5, ATG7, ATG10, ATG12, and ATG16L, are functional in the processing and ligation of LC3 to phagosomes.^147^ Therefore, LAP may represent a significant additional mechanism of phagocytosis and autophagy interaction.

Increasing evidence shows that the restraint of autophagy activation damages standard macrophage LAP. miR‐33 enhances lipid droplet accumulation in macrophages and inhibits apoptotic cell efferocytosis via LAP pathway. Recovery of autophagy and lysosomal biogenesis treatment improves efferocytosis and plaque necrotic areas using anti‐miR‐33.^132^ Besides, Liao et al.^148^ investigated that ATG5 ablation in macrophages exacerbate nicotinamide adenine dinucleotide phosphate oxidase expression and apoptosis in response to oxidative stress and ERS. Simultaneously, it makes apoptotic cells less visible to efferocytosis, which synergistically worsens plaque necrosis in advanced AS. However, whether LAP participates in the impairment of efferocytosis needs to be determined because AGT5 is a crucial component of LAP.

## 5 SIGNIFICANCE OF MACROPHAGE AUTOPHAGY IN CVDS

It is well established that autophagy plays a crucial role in controlling macrophage functions and states, which is also verified to play a regulatory role in CVDs. Appropriate macrophage autophagy can reduce pro‐inflammatory reactions and cardiovascular injury to improve the prognosis; hence, the modulation of macrophage autophagy may be a valuable approach for treating CVDs (Table 2).

**TABLE 2:** Application of macrophage autophagy in CVDs.

Category	Object	Mechanism	Effect on Disease	Reference
Atherosclerosis	LJP61A	Enhance M2 polarization via upregulation of two key autophagy upstream genes (*SIRT1* and *FOXO1*)	−	84
	Calycosin	Activate KLF2‐MLKL‐mediated autophagy to inhibit foam cell formation and apoptosis	−	159
	Melatonin	Inhibit NF‐κB activation and the nuclear localization of TFEB to ameliorate inflammation by macrophage autophagy	−	160
	Amino acid	Dampen mitophagy to induce dysfunction mitochondria‐induced apoptosis via mTOR activation	+	76
	Rivaroxaban	Ameliorate atherogenesis by scavenging the factor Xa‐PAR2‐mediated autophagy inhibition and subsequent inflammasome activity	−	161
	TAP	Facilitate plaque‐macrophage autophagy by trehalose, promote the reconstruction of endothelial barrier and regulate M2 polarization	−	162
	LOX‐1/RAP‐SNA	Induce robust autophagy and decrease the apoptosis of macrophages by RAP, and prohibit LOX‐1‐mediated foam cell formation	−	163
	Leuko‐Rap	Control mTOR‐SIRT‐NF‐κB‐mediated MMP activity, inflammatory cytokines and chemoattractant signals	−	117
Myocardial infarction	Metformin	Attenuate oxidative stress, inflammatory response and cardiomyocyte death through p‐AMPK‐mediated autophagy	−	43
	α7nAChR	Regulate mTOR‐related signalling pathway to inhibit pro‐inflammatory cytokine secretion	−	167
	miR‐144	Diminish the production of TNF‐β, SMAD‐3, MMP2, and MMP9 to ameliorate fibrosis by targeting mTOR	−	168
Heart failure and cardiac remodelling	HSF1	Induce upregulation of HIF‐1 expression to abrogate pressure overload‐induced heart failure	−	188
	Adiponectin	Increase the phosphorylation of AMPK to enhance autophagy and inhibit the NF‐κB pathway against hypertensive cardiac remodelling	−	191
	Cat S	Eliminate injured mitochondria to improve hypertension‐mediated cardiac remodelling	−	192
Cardiomyopathy	SIRT3	Deplete NLRP3 activation and endothelial malfunction by SIRT3‐ATG5 axis	−	198
Myocarditis	AR	Exert pro‐inflammatory effects by abolishing autophagy with reduced autophagosome formation and LC3‐II expression	+	202
Abdominal aortic aneurysm	Spermidine	Induce autophagy to limit aortic inflammatory cell infiltration and angiogenesis	−	208

Abbreviations: AMPK, AMP‐activated protein kinase; AR, androgen receptor; CVDs, cardiovascular diseases; HSF1, heat shock transcription factor 1; LAP, LC3‐associated phagocytosis; LC3, light chain 3; miR, microRNA; MMP2, matrix metalloproteinases‐2; mTOR, mechanistic target of rapamycin; NF‐κB, nuclear factor‐kappaB; RAP, autophagy activator rapamycin; SIRT, silent information regulator; SNA, spherical nucleic acid; TAP, Tr‐Arg‐PS; TFEB, transcription factor EB; TNF‐β, tumour necrosis factor‐β; ULK1, Unc‐51‐like autophagy‐activating kinase‐1.

### 5.1 Atherosclerosis

AS is the primary cause of CVDs and is characterized by foam cell formation and necrotic cores in plaques. Macrophages are pivotal effector cells that migrate to the inflamed sites of arteries to become cholesterol‐loaded foam cells and release various pro‐inflammatory factors. As a defence mechanism in AS, macrophage autophagy digests damaged proteins and accumulated lipids, and modulates inflammation in response to ROS, ox‐LDL, and ERS.

Plaque rupture and erosion eventually leads to atherothrombosis. Mechanical research has shown that SIRT6 diminishes contact with ECs in a macrophage autophagy‐dependent manner. It inhibits macrophage infiltration, inflammatory response, and occlusive thrombosis.^149^ Tissue factor (TF) is a coagulation and hemostatic modulator secreted by macrophages when the fibrous cap ruptures, which activates the clotting system and causes thrombosis. Knockdown of mTOR suppresses the expression of TF, MMPs, and monocyte chemoattractant protein (MCP)‐1 and promotes macrophage autophagy.^150^ Moreover, impaired monocyte autophagy jeopardizes the stability of atherosclerotic plaques, which may be related to the attenuated phosphorylation of AMPK.^151^, ^152^ ATG16L1 is reportedly expressed around the necrotic core of human carotid atherosclerotic plaques and co‐localizes with CD68 (a macrophage marker). ATG16L1 facilitates the production of pro‐inflammatory cytokines and MMPs, exacerbating plaque vulnerability.^153^

To stabilize plaques, VSMCs produce collagen to build fibrous caps. Canagliflozin increases VSMCs and exerts atheroprotective effects by enhancing macrophage autophagy.^154^ Apoptotic macrophages are exacerbated by MST1‐mediated autophagy inhibition, which secrete inflammatory factors to deteriorate the death of VSMCs, altogether contributing to plaque vulnerability.^155^ Furthermore, FURIN increases VSMC proliferation and lipid droplet translocation to arrest AS by the AMPK‐ULK‐PI3K pathway.^156^ Notably, chaperone‐mediated autophagy also takes part in AS development. This process is initiated early in atherogenesis but gradually decreases with disease progression, leading to a pro‐inflammatory state in macrophages and de‐differentiation of VSMCs.^157^, ^158^

Macrophage autophagy is a potential therapeutic approach for AS. SR‐BI regulates TFEB‐mediated macrophage autophagy to defend against atherogenesis by abrogating lipid accumulation, apoptosis, and inflammation.^40^ LJP61A reduces the M1/M2 macrophage phenotype ratio and suppresses lesion burden in AS, which provokes autophagic flux by upregulating the expression of SIRT1 and FOXO1.^84^ Research has demonstrated that calycosin, a flavonoid from Radix Astragali, helps resist macrophage inflammation and apoptosis. It enhances lipid hydrolysis and cholesterol efflux via KLF2‐MLKL‐mediated autophagy activation.^159^ Melatonin exacerbates macrophage autophagy and limits the inflammatory response to inhibit AS progression by galectin‐3 downregulation.^160^

mTOR is a potential target for the regulation of macrophage autophagy in AS. A high‐protein diet is a popular method of weight loss; however, it has been shown to raise the risk of AS. According to Zhang et al.,^76^ dietary proteins prevent the degradation of dysfunctional mitochondria via amino acid‐mTOR‐mitophagy‐mediated autophagy inhibition, which contributes to a rise in plaque complexity and caspase‐9‐mediated intrinsic apoptosis. Moreover, a recent investigation demonstrated that rivaroxaban administrates atheroprotection through abrogating PAR2‐mTOR‐mediated suppression of macrophage autophagy and obstructing inflammasome activation.^161^

According to the atheroprotective effect of autophagy activation, targeting macrophage autophagy in plaques has attracted increasing attention in recent studies. A type of NO‐driven carrier‐free Tr‐Arg‐PS (TAP) nanomotor, based on the reaction between trehalose (Tr, an mTOR‐independent autophagy inducer), L‐arginine (Arg), and PS, has been reported to prolong the circulatory half‐life and improve the drug utilization of Tr. To achieve the first‐phase targeting of the AS site, high expression of ROS and iNOS in the AS microenvironment is used as a chemoattractant, which interacts with Arg to generate NO as the nanomotor driving force to facilitate the chemotactic behaviour of the nanomotor. In the second‐phase targeting macrophages, the ‘eat me’ signal sent by PS is exploited to target macrophages to promote cell uptake in the plaques precisely, thus delivering Tr to macrophages using the step‐by‐step strategy. Subsequently, intercellular Tr can alleviate impaired autophagy in macrophages to constrict AS.^162^

In addition, Guo et al.^163^ constructed a RAP spherical nucleic acid (SNA) structure for plaque‐targeted delivery. Multiple hydrophobic RAPs are covalently grafted to the PS segment, designed at the 3′ terminus of the DNA strand, to form amphiphilic drug‐grafted DNA (RAP‐DNA), and consequently, self‐assembles into micellar SNA (RAP‐SNA). At the outer shell of the RAP‐SNA, the phosphodiester‐DNA segment can hybridize with LOX‐1 siRNA to form the drug‐co‐delivered SNA (LOX‐1/RAP‐SNA). This compound enhances macrophage autophagy by RAP‐mediated mTOR inhibition and prohibits the LOX‐1‐dependent formation of foam cells, thereby reducing the progression of AS and instability of atheromatous plaques. Utilizing the alterations in the endothelial monomolecular layer at the site of plaque formation, RAP‐loaded biomimetic nanoparticles (leukosomes) target inhibition of the mTOR‐SIRT‐NF‐κB pathway in macrophages of plaques to reverse vascular inflammation and plaque burden.^117^ Moreover, two novel macrophage‐targeted acidic nanoparticles developed by Zhang et al.^164^ have been shown to attenuate lysosomal dysfunction, autophagy retardation, and apoptosis activation in treating AS.

Although macrophage autophagy plays an essential protective role in AS, excessive autophagy activation can induce undesired results, such as apoptosis and sustained inflammation, which intensify pathological changes. Furthermore, regulation of macrophage autophagy may refer to the AS period. The large formation of ceroids is found in foam cell‐like macrophages isolated from late plaques. Its continuous degradation via autophagy increases the production of hydrogen peroxide and peroxidation of the lysosomal membrane, resulting in the uncontrolled release of lysosomal enzymes.^165^ In conclusion, further studies should discuss and investigate the optimum level of macrophage autophagy in atherogenesis and whether the effect of autophagy varies depending on the stage of AS.

### 5.2 Myocardial infarction

MI is a type of ACS with sudden blockage or occlusion of a major branch of coronary artery flow secondary to atherothrombosis, contributing to the blockage of cellular supply and ischaemic injury.^166^ Cheng et al.^151^ found that peripheral blood monocytes of patients with non‐ST‐segment elevation ACS and ST‐segment elevation MI have lower Atg7, Beclin1, and p‐AMPK levels, while having higher phosphorylated mTOR. An increasing number of studies have demonstrated that improving macrophage autophagy is advantageous for alleviating the prognosis of MI.

Metformin attenuates the release of pro‐inflammatory cytokines, infiltration of inflammatory cells, and NLRP3 inflammasome activation in macrophages to diminish the disarrangement of cardiomyocytes and infarct size in ischaemic myocardial injury. Further research found that metformin increases AMPK phosphorylation to upregulate autophagy function.^43^ Moreover, the alpha7 nicotinic acetylcholine receptor (α7nAChR) alleviates systemic inflammatory reaction and hypoxemic myocardium damage through mTOR‐related autophagy signalling in macrophages.^167^ miR‐144 is a cardioprotective miR, of which hypoxia depletes the endogenous level in the infarct zone. Macrophages, cardiomyocytes, and ECs take up intravenous miR‐144 to exacerbate autophagy by targeting mTOR. It reduces the production of TNF‐β, SMAD‐3, MMP2, and MMP9 to ameliorate fibrosis, left ventricle dilation, and heart failure (HF).^168^ Remarkably, Liu et al.^107^ found that inhibition of macrophage autophagy by Calhex231 retards myocardial fibrosis and cardiomyocyte necrosis, which is attributed to the constricted NLRP3 inflammasome activation.

Angiogenesis is a self‐regulating physiological process of tissue repair after MI accompanied by macrophage infiltration. M2‐type macrophages exert an anti‐inflammatory role and stimulate wound healing during recovery.^169^ They produce various pro‐angiogenic growth factors, such as MMP9 and vascular endothelial growth factor (VEGF).^170^ Salvianolic acid B boosts the expression of VEGF and subsequent neovascularization to evoke cardioprotective effects against MI by enhancing autophagy activity.^171^ However, intraplaque angiogenesis and haemorrhages are risk factors in plaque vulnerability and heart attack. SIRT6 abrogates TLR4 and HIF‐α secretion from macrophages through an autophagy‐mediated method, which abolishes intraplaque angiogenesis and stabilizes the plaques.^149^ Similarly, inhibition of mTOR counteracts circulating monocytes and neovascularization in plaques.^172^ Tongxinluo, a traditional Chinese medication, depletes inflammatory angiogenesis and macrophage apoptosis to stabilize vulnerable plaque by elevating Beclin‐1‐induced macrophage autophagy.^173^ Therefore, the role of macrophage autophagy in regulating blood vessel formation is not invariable, and should be applied according to the characteristics of different diseases.

Research has demonstrated that autophagy activation improves ischaemic myocardial growth and regeneration adjacent to ischaemic focal regions because salvaged amino acids are employed to synthesize new proteins.^174^ Macrophages facilitate neovascularization and cardiomyocyte proliferation owing to the timely resolution of inflammation.^175^, ^176^ Interference of NLRP3 by autophagy ameliorates I/R‐damaged cardiomyocyte regeneration.^177^ Disruption of inflammatory regression causes higher protease activity and jeopardizes recovery after MI.^178^ Additionally, it is reported that mammalian sterile 20‐like kinase (MST)‐1 may exert a crossover between macrophage autophagy and myocardial regeneration. MST1 inhibition elevates macrophage autophagy to restrain excessive inflammation and promote cardiomyocyte regeneration after injury.^179^ Thus, the macrophage autophagy‐mediated immune micro‐environment is crucial for cardiomyocytes with limited regenerative capacity.

### 5.3 Myocardial I/R injury

Javaheri et al.^180^ verified that macrophages subjected to hypoxia/reoxygenation stress exhibit a statistically significant decrease in LC3‐II, likely secondary to a relative deficiency in acidified lysosomes. This suggests that I/R injury is sufficient to provoke macrophage autophagy impairment. Although the effects of upregulated macrophage autophagy have not been reported in myocardial I/R injury, it has been confirmed to be protective in other organs, such as the liver and kidney.^181^, ^182^

Cardiomyocyte autophagy is controversial and dynamic during I/R. Cardiomyocytes exert defective autophagy and metabolism during ischemia. In contrast, they exhibit overactivated autophagy resulting from the heavy oxidative burden, which leads to excessive cell death during reperfusion.^183^, ^184^ For instance, aldehyde dehydrogenase 2 (ALDH2), an enzyme that catalyses the oxidation of aldehydes, induces cardioprotection through the paradoxical regulation of autophagy. It exacerbates autophagic flux by activating AMPK and inhibiting mTOR during hypoxia, while abrogating autophagy accompanied by the activation of the Akt–mTOR signalling pathway during reoxygenation.^184^ Similarly, ginsenoside Rb1 abolishes reperfusion‐induced over‐activation of cardiomyocyte autophagy by the PI3K‐Akt–mTOR pathway to alleviate myocardial I/R injury.^185^

Macrophages are the primary sources of oxidative stress during myocardial reperfusion. As a result, the ability of autophagy to reduce the oxidative burden and inflammation can contribute to the amelioration of myocardial injury during I/R. However, when regulating macrophage autophagy to defend against myocardial I/R injury, the dynamic changes in cardiomyocyte autophagy should be considered and undesirable effects on cardiomyocytes should be avoided. Macrophage autophagy‐targeted treatment has become a hotspot and more applicable approach for I/R injury.

### 5.4 Heart failure and cardiac remodelling

In addition to acute myocardial injury, elevated serum pro‐inflammation cytokines are detected in patients with chronic HF.^186^ Macrophages increase abundantly in the myocardium to promote the pathogenesis of HF via cytokine secretion. Moreover, the imbalance between primary cardiomyocyte death and macrophage‐mediated efferocytosis is a crucial trigger for the ongoing clinical deterioration of HF development. It results in chronic inflammation and collateral tissue injury.^187^ Macrophage autophagy can provide a method for HF anesis owing to the regulation of inflammation. The investigation shows that Heat shock transcription factor 1 (HSF1) alleviates cardiac malfunction and fibrosis in pressure overload‐induced HF. The autophagic flux of macrophages is activated with increased expression of LC3‐II, Beclin‐1, and autophagosome formation, associated with upregulated HIF‐1.^188^

Cardiac remodelling is a decisive factor in impaired ventricular function and the clinical course of HF. Macrophage accumulation has been implicated in cardiac remodelling and contributes to fibrosis through TGF‐β production.^189^ In ATG5 haplodeficient mice, macrophage‐mediated ROS production and inflammatory response elevate cardiac fibrosis and inflammation.^190^ Thus, activation of macrophage autophagy also plays a protective role in ameliorating cardiac remodelling. Adiponectin, a 30‐kDa adipokine secreted by adipocytes, exerts anti‐inflammatory effects and deters fibrosis against hypertensive cardiac remodelling. Adiponectin can mitigate the expression of pro‐inflammatory cytokines in an autophagy‐dependent manner. By phosphorylating AMPK, adiponectin induces macrophage autophagy and inhibits NF‐κB activation.^191^ In addition, Cathepsin S‐deficient mice show robust production of pro‐inflammatory cytokines and severe cardiac fibrosis, attributed to the abnormal accumulation of autophagosomes in macrophages. Impaired autophagy blocks the elimination of damaged mitochondria, thus leading to ROS production and NF‐κB activation to exacerbate hypertension‐mediated cardiac remodelling.^192^

### 5.5 Metabolic cardiomyopathy

With the clinical features of cardiac hypertrophy and diastolic dysfunction, metabolic cardiomyopathy develops in the context of chronic pathological metabolic dysfunction.^193^ Excessive nutrients trigger enormous inflammatory signalling pathways. The characteristics of early‐stage cardiomyopathy include metabolic disturbance‐induced subcellular low‐grade inflammation and subsequent component abnormalities, such as ERS and mitochondrial dysfunction. While cardiomyocyte injury and fibrosis with impaired myocardial structure and function contribute to a vicious cycle of inflammatory cell infiltration and neurohumoral activation in advanced stage.^42^

Macrophages migrate into the heart by intramyocardial chemokines and secret pro‐inflammatory cytokines, such as IL‐1β, TNF‐α, and MCP‐1.^194^ Free fatty acids are capable of activating macrophages to potentiate inflammatory response and insulin resistance via IL‐1β‐mediated islet β‐cell dysfunction.^195^ Upregulated expression of M1 polarization in the myocardium leads to the inflammatory micro‐environment and activation of metabolic cardiomyopathy‐related pathways.^196^ These findings indicate that macrophage‐induced inflammation takes part in the pathogenesis of metabolic cardiomyopathy.

Notably, metabolic diseases impede autophagy and mitophagy with a reduction in AMPK activity, resulting in oxidative stress and NLRP3 inflammasome activation in macrophages.^197^ Furthermore, the generation of toxic lipid intermediates and dyslipidemia are accompanied by the onset of cardiomyopathy.^193^ Considering that autophagy is insufficient in metabolic cardiomyopathy and its function in affecting inflammation and lipid metabolism, we suspect that the upregulation of autophagy activation may play a dominant role in treating cardiometabolic syndrome. SIRT3‐mediated autophagy ameliorates diabetic cardiac dysfunction. It can impede NLRP3 inflammasome activation and IL‐1β to ameliorate hyperlipidemia‐induced endothelial dysfunction. SIRT3 overexpression of macrophage abates the protein levels of α‐SMA, collagen‐I, VACM‐1, and intercellular adhesion molecule 1 in ECs by SIRT3‐ATG5‐IL‐1β axis.^198^

### 5.6 Myocarditis

Myocarditis is an inflammatory disease of the myocardium and is pathologically ruled out by pronounced macrophage infiltration and cardiomyocyte death.^199^ On account of its high plasticity and pathophysiological role, the modulation in macrophages provides a new approach to myocarditis therapy.^200^ For example, miR‐30a‐5p silencing skews the macrophage subset into M2 and suppresses the inflammatory response to ameliorate viral myocarditis (VMC) injury.^201^ Autophagy, as a regulator of macrophage function, may play a protective role in myocarditis. Androgen receptor (AR) inhibition exerts anti‐inflammatory effects to promote tissue repair in an experimental autoimmune myocarditis (EAM) mouse model. Further investigation showed that AR suppression alleviates the secretion of pro‐inflammatory cytokines and the M1/M2 polarization rate via enhancing autophagy in macrophages. This is corroborated by the increased LC3‐II/I ratio and autophagosome formation, thus diminishing apoptotic injury and tissue fibrosis in EAM.^202^

It is worth noting that coxsackievirus B3 (CVB3), one of the major pathogens of VMC, can increase cellular autophagosome formation to support its replication in the host and release it from cells depending on apoptosis.^203^ Autophagy is not enhanced because there are no apparent changes in autophagic adaptor p62 expression after CVB3 infection. However, activation of autophagosome–lysosome fusion can abrogate viral proliferation. It suggests that suggesting that lysosomal fusion is commendatory and autophagosome formation should be avoided when treating CVB3‐infected viral myocarditis.^204^

### 5.7 Abdominal aortic aneurysm

The histopathological features of abdominal aortic aneurysm (AAA) are chronic inflammation and production of oxidative stress.^205^ In particular, macrophage infiltration of the aortic wall leads to the sustained local and systemic inflammatory response in AAA.^15^ Secretion of IL‐1β, especially by macrophages, has been documented to be elevated in aneurysmal tissue.^206^ Studies have found that macrophage autophagy is activated by AAA‐related factors, thus postponing the AAA process.

Angiotensin‐II, a principal mediator peptide of the renin‐angiotensin system, can mediate adverse vascular remodelling and dysfunction in AAA through ROS generation and inflammation. These processes can stimulate macrophage autophagy and are mitigated by protective mitophagy.^207^ As a result, upregulation of macrophage autophagy may be a candidate method for suppressing and treating AAA. Liu et al.^208^ demonstrated that spermidine markedly limits aortic inflammatory cell infiltration, angiogenesis, and neutrophil circulation in an AAA mouse model by accumulating autophagosomes in medial VSMCs and adventitial macrophages. Moreover, naringenin has been shown to effectively reverse the progression of established AAA by targeting the macrophage TFEB‐14‐3‐3 epsilon interface. Naringenin can directly bind to 14‐3‐3 epsilon to inhibit TFEB‐14‐3‐3 epsilon interactions, and subsequently promote the induction of TFEB and autolysosome formation in macrophages. TFEB‐dependent inhibition of NLRP3 inflammasome and encouragement of GATA3‐IRF4‐STAT6‐mediated reparative M2 phenotype synergistically exert beneficial roles in abolishing AAA.^209^ Notably, TEFB is a vital regulator of autophagy‐related genes. Further experiments are needed to clarify whether TEFB‐dependent modulation of inflammasomes and polarization of macrophages are associated with autophagy to hinder inflammation in aortic tissue.

## 6 DISCUSSION AND PERSPECTIVES

In summary, immune cell‐mediated dysfunction of inflammation participates in the development and pathophysiology of CVDs. Macrophages play a predominant role in the initiation and deterioration during the whole progression of diseases. Autophagy is a highly conserved mechanism of self‐protection that degrades old organelles and abnormal protein aggregates for renovation and homeostasis. Recent studies have demonstrated that autophagy extensively modulates macrophage functions by eliminating inflammatory particles and autophagy‐related protein‐mediated signalling pathways. They also provide evidence that autophagy activation can attenuate monocyte recruitment, monocyte–macrophage differentiation, proliferation ability, and cell death to control the number of macrophages in CVDs. In addition, macrophage autophagy has an anti‐inflammatory effect on CVDs by decreasing inflammasome activation, pro‐inflammatory cytokine and chemokine production, and polarization toward the M1 phenotype. Moreover, macrophage autophagy can regulate metabolism via complex pathways involving crosstalk with lipids, the accumulation of which contributes to foam cell formation and necrotic cores in atherosclerotic plaques. Autophagy also regulates macrophage phagocytosis to reduce vascular injury. Therefore, modulation of autophagy in macrophages via affecting these parameters represents a potential and desirable approach for CVD treatment.

However, the conclusions above are based on a moderate level of macrophage autophagy, because overactivated autophagy escalates inflammation and adverse effects in CVDs, implicating two sides of autophagy. Although knowledge of the impact of autophagy on macrophage regulation has evolved and expanded, several open problems remain to be addressed regarding the molecular mechanisms underlying the modulation of autophagy in macrophage functions and the continuous change in macrophage autophagy in the context of CVDs, in addition to the value of macrophage autophagy as a target for CVD therapies. Remarkably, studies have indicated the cardioprotective role of macrophage autophagy in some CVDs, such as AS, MI, cardiac remodelling, and myocarditis. Nevertheless, more disease models are required to corroborate the effects of macrophage autophagy in CVDs. The present findings are mainly based on in vitro experiments. Further studies are required to validate the beneficial results in vivo by targeting macrophage autophagy in CVDs, and to determine whether they are applicable to humans. Nevertheless, an increasing understanding of its mechanisms and functions predicts that an in‐depth hypothesis regarding macrophage autophagy may provide a proof‐of‐concept of a novel theoretical target for CVD treatment.

## AUTHOR CONTRIBUTIONS

All the authors have read and approved the final review article. Hongxia Li, Tinbo Jiang, Tianke Yang, Wanqian Pan, Jun Zhang, and Lei Zhang conceived and wrote the review. Yiyi Song, Mingyue Tan, and Yunfei Yin searched and analysed the literature. Tianke Yang, Yiyi Song, Yue Zhang, and Lianhua Han reviewed and edited the review. Hongxia Li, Tinbo Jiang, Tianke Yang, and Wanqian Pan modified this review.

## FUNDING INFORMATION

This work is supported by grants from the National Natural Science Foundation of China (Nos. 81770327 and 81100173).

## CONFLICT OF INTEREST STATEMENT

The authors have declared that no competing interest exists.

## Data Availability

Data sharing is not applicable to this article as no new data were created or analysed in this study. Figures were created by Biorender.
